# Why Turkish Parents Refuse Childhood Vaccination? A Qualitative Study

**DOI:** 10.34172/aim.2023.41

**Published:** 2023-05-01

**Authors:** Salih Aslan, Adem Ozkara, Ismail Kasım, Hilal Aksoy

**Affiliations:** ^1^Evren Integrated Health Center, Ankara, Türkiye; ^2^Department of Family Medicine University of Health Sciences,, Ankara City Hospital, Ankara, Türkiye; ^3^Department of Family Medicine, Hacettepe University Faculty of Medicine, Ankara, Türkiye

**Keywords:** Antivaccination movement, Parents, Infectious diseases

## Abstract

**Background:**

Anti-vaccination is spreading among parents. In 2017, 23000 families in Turkey refused vaccinations for their children. Meanwhile an increase in infectious diseases was observed, which might be caused by vaccination rejection. The reasons why families do not vaccinate their children may be very different, such as side effects, or advocation for "healthy life" by gurus. However, the real reasons for vaccine refusal are unknown. Our aim is to determine the reasons for anti-vaccination in Turkey.

**Methods:**

In order to reveal the real reasons for not taking the vaccine, we planned to conduct interviews with the representatives of the vaccine rejection group using qualitative research methodology with the "grounded theory" method. We searched some anti-vaccination blogs to find candidates for interviews. Within the scope of our study, parental concerns about vaccinations were classified by analyzing the data obtained from semi-structured questions and interviews recorded with voice recorders in face-to-face interviews with 21 parents in 13 cities of Turkey.

**Results:**

The obtained findings were classified under the headings of ‘’mistrust’’, ‘’vaccine efficacy-importance’’, ‘’decision-making processes - bases’’, and ‘’law-ethics’’. Mistrust was the main theme, almost singularly, as the most important reason for vaccine rejection. The salient reasons for mistrusts were: Companies which produce vaccines especially international companies because of conspiratory beliefs; health authorities, because of the belief about non-transparency in epidemiologic data, immunization council etc. and healthcare professionals, because of their non-communicative and non-concerned attitude.

**Conclusion:**

Mistrust is hard to overcome. The beliefs of the patients cannot be easily changed. As a result of our study, we made some recommendations for health authorities, healthcare professionals, companies and other related stakeholders.

## Introduction

 Infectious diseases are one of the major public health problems in the world, especially in the developing and underdeveloped countries.^1^ Some infectious diseases cause outbreaks, causing serious mortality and morbidity, creating a significant burden for the economies of countries and public health. These diseases, which have caused millions of people to die or become disabled throughout history, are thought to be largely caused by socioeconomic-cultural and environmental factors.^2,3^

 By vaccination, it is aimed that the person to whom a microorganism or product obtained from the microorganism is administered, by imitating natural infection, will produce the appropriate immune response to that infection and thereby be protected against infection.^4^ In vaccination practices, the primary target is to prevent disability and death.^5,6^

 Vaccines, which have been developed as a protective tool against some infectious diseases and included in the countries’ immunization programs, are one of the subjects that medical science has been researching and discussing the most. Parents have no tolerance for the side effects observed due to the vaccine given to healthy people, especially healthy babies, and this causes the parents to be disturbed and question the vaccines.^7^

 Some serious side effects reported after vaccinations administered to the healthy individuals and thought to be related to the vaccine, have attracted public attention. Vaccines are medicinal products and the World Health Organization (WHO) has recommended monitoring the side effects of vaccines included in all countries’ immunization programs.^7,8^

 “National Immunization Programs”, which differ across countries, are affected by many factors such as epidemiology of diseases, problems caused (morbidity, mortality, frequency, costs etc), characteristics of vaccines (reliability, efficacy, accessibility, etc.) and scientific research. While countries create immunization programs, the recommendations of organizations such as the WHO can be also effective.^9^

 Attitudes and beliefs, including mistrust of medical and scientific elites, resistance to government authority, and adherence to “natural” or alternative health belief systems are believed as reasons for refusal.^10^ But these reasons can vary across countries and religions.^11^

 Confusion about vaccination is based on very different reasons. As can be seen from the studies listed in Table 1, high rates of vaccine hesitancy and vaccine rejection are seen in different countries.^12-24^

**Table 1:** Reasons For Vaccine Hesitancy

**Author**	**Year**	**Type of Vaccination**	**Sample**	**Population**	**Refuser %**	**Hesitant %**	**Concerns**	**Reference**
Lin et al	2020	COVID-19	3541	Chinese	4.5%	66.7%	√ Lack of adequate information. √ Foreign-made (origin of vacc.) √ Vaccine efficacy. √ Adverse event.	^12^
Reuben et al	2020	Childhood	484	USA, Canada, United Kingdom parents	N/A	N/A	√ Mistrust in medicine. √ Mistrust in physicians.	^13^
Palamenghi et al	2020	COVID-19	1972	Italian	41%		√ Mistrust in science. √ Hesitancy about vaccines efficacy.	^14^
Barello et al	2020	COVID-19	934	Italian university students	13.9%		N/A	^15^
Yalçin et al	2020	Childhood	177	Turkish health workers	N/A	N/A	√ Questions about vaccine safety. √ Mistrust in health system. √ Anti-vaccine publications in social media. √ Lack of information about national vaccination schedule and components of vaccines.	^16^
Rumetta et al	2019	Childhood	14	Malay vaccine refusers	N/A	N/A	√ Lack of confidence in modern medicine and health care workers. √ Pharmaceutical conspiracy to sell medicines. √ Preference of natural approach to health. √ Personal instincts. √ Religious beliefs. √ Negative effects and content concerns of vaccines. √ Doubt of necessity. √ Lack of information and knowledge regarding vaccines.	^17^
Bianco et al	2019	Childhood	575	Italian parents	N/A	7.7%	√ Receiving information from mass-media. √ Lack of trust regarding information about vaccines. √ Having been discouraged by the pediatrician. √ Belief in "vaccinations are primarily an economic business of pharmaceutical companies."	^18^
Dubé et al	2016	Childhood		Canada Canadian Immunization Research Network	3	19	√ Safety and/or effectiveness of vaccines. √ Logistics and accessibility barriers. √ Religious or conscientious exemptions. √ Negative and false information online and in social media.	^19^
Santibanez et al	2018	Childhood	36 184	USA, Parents of children (National Immunization Survey- NIS)	N/A	25.8	√ The number of vaccines, side effects, knowing anyone who has had a serious long-term side effect. √ Not considering the child’s doctor or health provider as the most trusted source of information about childhood vaccines	^20^
	2019	Childhood	39 617		N/A	19.5		
Dror et al	2019	COVID-19		Israel healthcare employees and general population	25	N/A	√ Fears about vaccine’s safety, quality control, side effects, doubted efficiency. √ Wait until tested by others. √ Associated COVID-19 illness. √ Physiological immunity better. √ Pregnancy	^21^
Kennedy	2019	Childhood	65 819	European	N/A	N/A	√ Anti-establishment politics. √ Vaccines are not important, effective and safe	^22^
COCONEL Group	2020	COVID-19	1012 COCONEL Survey	French	26	N/A	√ Political views √ Vaccine safety √ Intransparent policy	^23^
Salali and Uysal	2020	COVID-19	3936	Turkey	3	31	√ Beliefs on the origin of the novel coronavirus (natural/artificial) √ Gender. √ Risk perception. √ Anxiety level. √ The degree of satisfaction with government’s response to the pandemic	^24^

N/A, Not applicable.

 It is a known fact that vaccine refusal has been associated with outbreaks of invasive *Haemophilus influenzae* type b disease,^25^ varicella,^26^ pneumococcal disease,^27^ measles^28^ and pertussis.^29^ Often, these outbreaks occur in communities with high rates of vaccine delay and refusal.

 Even during the COVID-19 outbreak, there are people who do not want to receive vaccines. In a study conducted on 32,361 adults overall, 14% of participants reported unwillingness to receive a vaccine for COVID-19, whilst 23% were unsure.^30^

 Vaccines, which the science of medicine defines as the most effective tool in the fight against infections, are also one of the topics that are increasingly being questioned and discussed. The WHO released its top 10 threats to global health in 2019 and vaccine hesitancy is at the eighth order.^31^

 The Strategic Advisory Group of Experts (SAGE) is the principal advisory group to the WHO for vaccines and immunization. SAGE recommends every country to assess their own vaccine refusal and hesitancy reasons and report them,^32^ so that we can understand our patients better and communicate with them more easily. Qualitative studies are types of researches that investigate the causes of a problem in depth in the context of grounded theory. In our study, we aimed to determine the reasons for childhood anti-vaccination in Turkey using this method.

## Sampling

 In our country, which we think may be suitable for sample selection, we considered the social media groups that are followed by the parents who partially or completely reject vaccinations or who are interested in this subject. Facebook groups on anti-vaccination were investigated. In the social media, groups with about 50 000 followers, a large number of messages were examined, including parents’ experiences and thoughts on vaccines.

 By contacting the editor of one of the social media groups in our country, information was provided about the purpose and method of our study, and then we published in the social media group an announcement on the purpose and method of our study, as well as our desire to reach volunteer participants.

 After the announcement, approximately 200 parents sent a message stating that they would like to voluntarily participate in our work. We formed a close social media group for parents who sent messages. We asked parents to express their thoughts, concerns about vaccines and where they live via private message (without writing to the group) in short and main headings.

 Approximately 70 sent messages were analyzed and the concerns (reasons for vaccine refusal) were classified. Two separate classifications were made as “reasons according to the participant candidates” and “participant candidates according to the reasons”.

 In this present study done, we tried to select the smallest possible number of parents, taking into account the number of cities to be traveled to. We chose 20 participants, but 5 of them withdrew from participating in the study for various reasons like lack of time, unwillingness and illness during the process of making a travel plan to reach the parents. We reached 15 participants.

 “Snowball sampling” was also used to reach the participants. Six participants were interviewed in this way. Collecting additional data was stopped when we believed that no additional information could be obtained.

###  Data Collection 

 The study was conducted between June 2017 and May 2018. Verbal and written informed consent was received from each participant for participation and audio recording of the interviews. The research participants were interviewed using semi-structured questions in approximately 20- to 150-minute interviews. There was no time constraint. All interviews were recorded digitally, and the records were transcribed exactly, including all emphases and special expressions. Body language, major gestures, and facial expressions were evaluated during the interview and noted by the interviewer. While transcribing the interview, all special expressions were stated in parentheses, with special notes on emotional content. There were no repeated interviews.

###  Data Analysis

 Data were analyzed using a thematic framework method.^33^ This method was chosen because it is the most appropriate tool for supporting thematic (qualitative content) analysis; it provides a systematic model for managing and mapping the data.^34^ The stages of the framework method were followed, but matrices were not constructed.^35^ Transcriptions of the interviews were read and analyzed by two researchers separately.

## Results

 Within the scope of the study, a total of 21 parents were interviewed face-to-face in 12 cities (Antalya, Aydın, Mugla, Usak, Izmir, Bursa, Balıkesir, Canakkale, Ankara, Istanbul, Samsun and Mardin) in Turkey.

 The findings we obtained from the research were classified under 4 main themes:

1. Mistrust
2. Importance-effectiveness of the vaccines
3. Decision Making Process-Bases
4. Law and Ethics

The themes and major categories under these themes are given in Table 2.

**Table 2:** Reasons for Anti-vaccination

**Themes**	**Major Categories**
Mistrust	Contents of vaccines Side effects of vaccines Scientists and scientific studies Domestic vaccine production
Importance-effectiveness of the vaccines	Unvaccinated child is thought to be healthier The notion that vaccines are unnecessary
Decision making process-bases	Social environment Experiences
Law and ethics	Lack of informed consent Mandatory vaccination

###  Mistrust 

 Mistrust was the main theme that was alone among the most important reasons for vaccine rejection.

 Negative thoughts about institutions such as vaccine manufacturers, global vaccination policy, the CDC, and the WHO constituted an important part of the problem of trust related to vaccines.

 “*Anti-vaccination also comes from mistrust in the pharmaceutical industry. They will make them sick so that they can give them new drugs. Such a vicious circle.’’ *(Number: 9)

 “*There (Africa), the most needed material is water, well! Why don’t you study that but you pay so much attention to this vaccine? The people who are planning population control around the world are also the guys who carry out and produce vaccination programs.’’ *(Number: 17)

 The fact that vaccines are imported and not produced in our country is one of the reasons that vaccines are not trusted.

 “*The lack of vaccine production technology in Turkey and the vaccines are imported. Why didn’t Turkey invest in vaccine production? Why did we give up on the ones we were able to produce?” *(Number: 3)

 Mistrust was also expressed in the form of the idea that imported vaccines were not adequately controlled, that the cold chain could not be preserved and the negative problems associated with the protection of the cold chain.

 The idea that scientific studies and scientists may be biased is one of the reasons for mistrust of vaccines, as expressed by some of our participants.

 It was stated by most of our participants that the substances contained in the vaccines and used in the production phase are harmful. Some of our participants stated that some animal additives used in their content and production phase are against their religious beliefs.

 “* We know what the mercury in vaccines causes.’’ *(Number: 9)

 The majority of our participants stated their opinions about the side effects of vaccines, while some of our participants stated their experiences and observations about the side effects of the vaccines. These side effects included frequent illness, high fever, developmental retardation, anorexia, allergic side effects, autoimmune diseases, autism, behavioral disorders such as attention deficit etc., nervous breakdowns, sleeping disorders, encephalitis, neuritis, convulsion, sudden infant death, infertility, and post-vaccination paralysis.

 Most of our participants expressed their opinions and objections regarding the timeline of vaccination.

 “*We damage the immune system by vaccinations until the age of 2.’’ *(Number: 1)

 “*Let a child complete their immune system and its development. A child is affected at the minimum level after a certain age or becomes tolerant.’’ *(Number: 3)

###  Importance-Effectiveness of the Vaccines 

 The vast majority of our participants had negative thoughts on the effectiveness, importance and necessity of the vaccines.

 “*Despite being fully vaccinated, (her child) got mumps. So I think vaccines do not protect much. I thought to myself that these vaccines are not protective enough.’’ *(Number: 2).

 Most of our participants thought that unvaccinated children are healthier and get less infections.

 “*My daughter, who did not get vaccinated, was able to eat everything, but the allergies of her friends were still very severe and increased after each vaccine.’’ *(Number: 4).

 Most of our participants stated that the vaccines are partially or completely unnecessary.

 “*Chickenpox is not a fatal disease, it is a disease we all have experienced. Chickenpox vaccine is unnecessary.’’ (Number:2) *

 “* I don’t care about the rotavirus… hepatitis A is unnecessary.’’ *(Number: 3).

###  Decision Making Process-Bases 

 The process by which our participants changed their opinions about vaccines differs. Some of our participants stated that they did research about the vaccines due to the problems they experienced in their first children, and that they decided not to vaccinate their subsequent children as a result of their research. Others were unaware of the discussion issues related to the vaccines in the beginning, they reported that they were partially or completely opposed to vaccines through the internet and especially the posts on social media.

 “*Due to my own researches and impressions, I didn’t need fatwa and I just didn’t get my child vaccinated and that’s it. Unless there is a risk and while I am not sure about its content yet, I’m against all the vaccines.’’ *(Number 8).

 “*I didn’t know much about the vaccinations before. You start searching unknowingly and incredible doors open up in front of you. After doing some research on the vaccines, we ask our physician biasedly. So we say that we do not want to have it done, why should we have it done?’’ *(Number 10).

 Social circles, the internet, newspaper and television news, the domestic and foreign websites they follow, the social media groups and the posts made in these groups also played an important role in the formation of negative opinions in our participants about the vaccines.

 Some parents disagreed about the decision not to get vaccinated, while others supported each other.

 “*Actually, I don’t care about the rotavirus much. We could not agree with my wife very much in her decision to get it done.’’ *(Number: 3)

###  Law and Ethics 

 In our interviews, the opinions of most of our participants about the healthcare professionals and the physicians, what happened in the health centers related to the subject of vaccination, the attitudes of the healthcare professionals towards them because they did not get vaccinated, and their views or observations about the vaccine from the physicians around them were expressed in different ways.

 “*I called my child’s physician, I said, the vaccine is badly affecting my child now, I’m sure the vaccination did this. The physician says no there can’t be such thing, I called the midwife of the health center and she says the same! This vaccine causes such an effect on my child. Have you taken a note about me, have you filled any documents? Based on what do you say no to me?’’ *(Number: 4).

 “*The physicians cannot give clear information, they do not mention the side effects. They never talk about the sudden deaths. I would believe them if they could explain it a little bit. They cannot provide statistical information about the benefits of the vaccines. My family physician is actually good, but I’ve never been enlightened on these issues.’ *(Number: 7).

 The parents expressed their thoughts on the legal process and the mandatory vaccination application since they did not accept the vaccinations.

 “*Forced vaccination is wrong.’’ *(Number:1)

 “*If you want to do something to a person who does not want that thing, you must have very serious and solid reasons. I say it’s not 100% reliable, it’s not 100% protective, it can be the cause of many diseases. While there is so much suspicion about the vaccines, mandatory vaccination does not make sense.” *(Number:16).

## Discussion

 In our country and also in the world, parents have concerns for many different reasons related to vaccines and immunization policies, which are being discussed and questioned with increasing momentum. This study is the qualitative evaluation of vaccination in Turkey from the public’s point of view and is expected to fill an important knowledge gap in the field of medical care. We accept that this study also has certain limitations. One could also argue that the number of participants we included (n = 21) was low. However, we stopped including additional interviewees when we felt that we had reached saturation and when no further information could be obtained from additional interviews. Finally, our results cannot be generalized to all anti-vaccine parents but the obtained data remain valuable because they provide a good picture of their thoughts and beliefs.

###  Comparison with Existing Literature 

####  Mistrust

 Mistrust is one of the leading reasons for almost all parents to partially or completely reject vaccines and have hesitations about vaccines.

 There are many studies in the scientific literature on the content and side effects of the vaccines. Most of these studies have been conducted about the mercury and aluminum content.^36,37^ There are also publications in the literature about the autoimmune diseases reported after the vaccinations.^38^ There are many studies on the relationship between vaccination and autism. However, different results are reported even in meta-analysis studies with high evidence value.^39^ Parents who read only the literature about the negative features of the vaccines might be insecure about them because they do not compare.

 Mistrust about the pharmaceutical industry is a major cause of concern for our participants regarding all imported vaccines and our immunization policies, which are included in our national vaccination program. Mistrust in the pharmaceutical companies and their employees is not only a matter for the parents with hesitations about vaccines in our country. The report published by the European Center for Disease Prevention and Control in 2015 showed that the healthcare professionals do not trust the pharmaceutical companies that do not give enough information about the side effects due to their financial interests and try to put the physicians under pressure.^40^

 In studies funded by the pharmaceutical industry, the probability of results in favor of sponsor company’s product is higher than those funded by other sources, causing prejudices about the results of published research funded by the pharmaceutical industry.^41^

 The fact that there are insufficient scientific studies that can illuminate the controversial issues related to the vaccines in our country, the scientific studies carried out abroad and the opinions that scientists conducting these studies may be biased are the findings we reached in our study as the reasons that are at the forefront of parents’ concerns about vaccines and our immunization policies.^42^

 Since necessary investments have not been made in our country, which started vaccine production from the first years of the Republic and could produce its own vaccines until the end of the 1990s, all vaccines are being imported for about 20 years.^43^ Despite the news about the resumption of domestic vaccine production from time to time, there is limited achievement in domestic vaccine production.^44-46^

 The vaccines are commercial products and also have a profitable market. While the value obtained from the vaccine trade in 2001 was 6 billion dollars, it is estimated that a total of 30 billion dollars sales will be made in 2020.^47^ Our vaccine expense was 18 million TL (US$ 126 million) in 2002 and approximately 883 million TL (US$ 6.181 million) in 2016 in our country.^48^

 Having national vaccine production technology is one of the important biotechnology policies. Vaccines are a strategic product for which foreign dependency cannot be accepted and vaccine production is a public responsibility. Therefore, domestic vaccines should be produced in our country and necessary steps should be taken to have vaccine production technology.^49^

 Most of the parents who participated in our study stated that they do not trust the vaccines because they are imported and if they are produced in our country, they will trust the local vaccines more. They think some vaccines must have a “halal certificate” in order to produce more confidence and having national vaccine production opportunities is closely related to our national security. In this sense, the steps to be taken by the Ministry of Health, especially in local vaccine production, are very important and they concern our national security closely. In our country, Turkish Medicines and Medical Devices Agency within the Ministry of Health is the institution in control of imported vaccines.^50^

 Some parents believe that there are some problems with the maintenance of the cold chain and the inspections are not sufficient and appropriate in the health centers. Cold chain is a system consisting of people and materials that protect the effectiveness of a vaccine from its production until its administration to a person and ensures that sufficient amount of effective vaccine is available to those in need. With the Ministry of Health’s Extended Immunization Program Circular, issues related to the protection of the cold chain were specified, and the duties of the cold chain officer and the vaccine officer were separately defined at the provincial, district and affiliated health center levels.^6^ With the Vaccine and Anti-Serum Cold Chain and Stock Tracking System (ATS), which has been implemented since 2015, the temperature information of the cold storage, vaccine cabinets where vaccines and anti serums are kept is monitored instantly and regularly; necessary measures are taken when the temperature measurements are determined to be outside the specified limits, and the authorities are warned to take necessary measures.^51^

####  Importance-Effectiveness of the Vaccines

 Parents’ doubts about the benefits and necessity of the vaccines are also an important factor in their partial or complete opposition to vaccination. According to the results of a study conducted in the United States in 2009, 25.8% of the American parents who have 24-35-month-old children, delayed the vaccinations (did not get vaccinations in time), 8.2% refused to vaccinate their children and 5.8% both delayed the vaccination and refused to get vaccines. The study highlights that most parents who delay or oppose vaccination have doubts about the benefits of the vaccines.^52^

####  Decision Making Process-Bases

 The Immunization and Advisory Board within the Ministry of Health, which has a very important position and function, makes advisory decisions in the determination of our country’s immunization policies, as stated in the Ministry of Health’s Extended Immunization Program Circular.^6^

 One of the main safety concerns of the parents who participated in our study was the idea that vaccines do not have a very high safety profile that will leave doubts as they are administered to completely healthy babies/people in order to protect them from some possible infectious diseases in the future. Also, concerns and doubts about vaccines are present among healthcare professionals.^53,54^ Some vaccines can be withdrawn by the regulatory agencies due to the problems experienced after they are used on people, and the vaccine manufacturer company can stop the administration.^55^

####  Law and Ethics

 Vaccines have been one of the most discussed topics for a long time.^56^ Discussions on the ethical issues brought by the public health policies associated with the vaccines cover many issues beyond individual vaccine decisions. Public health policies related to vaccines have brought with themselves many ethical issues that have been the focus of recent discussions.^57^

 Healthcare professionals, as with any medical intervention, should have sufficient knowledge to enlighten the parents about the causes, content, side effects, protection of the vaccines and the risks they may encounter in the absence or administration of the vaccines.

 The other important subject is to distinguish between vaccine hesitancy and vaccine refusal. The WHO defines vaccine hesitancy as a “delay in acceptance or refusal of vaccines despite availability of vaccination services.”^58^ Vaccine-hesitant parents are generally in the middle of a spectrum and underimmunize their children instead of not immunizing them at all.^59^ Parents who had been informed about vaccines by physicians were less likely to have vaccination concerns compared with those who received information from other people.^60^ Therefore, family physicians can play an important role in counseling vaccine-hesitant parents and also the public.

 The parents in our study were distrustful about the vaccines and the immunization policies for many different reasons. Our findings show that the distrust as the main theme was almost the most important reason for parents’ concerns about vaccinations and immunization policies.

 In a recent study that was conducted in Turkey to explore healthcare professionals’ observations about the parents’ decisions to reject vaccination, healthcare professionals suggested some interventions which were especially related to overcoming mistrust and lack of knowledge.^21^ This shows that health care professionals are aware of reasons for vaccine refusal.

## Conclusion

 There is a need for scientific studies to understand the reasons for vaccine rejection and to clarify controversial issues related to the vaccines. Especially, the reasons for vaccine hesitancy should be clarified in future studies. It is easier to change the opinion of vaccine hesitant individuals than vaccine refusers. So, health care professionals, managers and decision makers should understand people more deeply and produce more specific solutions.
